# Novel Anti-Cytokine Strategies for Prevention and Treatment of Respiratory Allergic Diseases

**DOI:** 10.3389/fimmu.2021.601842

**Published:** 2021-05-18

**Authors:** Ekaterina O. Gubernatorova, Olga A. Namakanova, Ekaterina. A. Gorshkova, Alexandra D. Medvedovskaya, Sergei A. Nedospasov, Marina S. Drutskaya

**Affiliations:** ^1^ Engelhardt Institute of Molecular Biology, Russian Academy of Sciences, Moscow, Russia; ^2^ Lomonosov Moscow State University, Moscow, Russia; ^3^ Center for Precision Genome Editing and Genetic Technologies for Biomedicine, Engelhardt Institute of Molecular Biology, Russian Academy of Sciences, Moscow, Russia; ^4^ Department of Immunobiology and Biomedicine, Sirius University of Science and Technology, Sochi, Russia

**Keywords:** anti-IL-6, anti-TNF, anticytokine therapy, severe asthma, combined cytokine targeting, alarmins in asthma

## Abstract

Asthma is a heterogeneous inflammatory disease characterized by airflow obstruction, wheezing, eosinophilia and neutrophilia of the airways. Identification of distinct inflammatory patterns characterizing asthma endotypes led to the development of novel therapeutic approaches. Cytokine or cytokine receptor targeting by therapeutic antibodies, such as anti-IL-4 and anti-IL-5, is now approved for severe asthma treatment. However, the complexity of cytokine networks in asthma should not be underestimated. Inhibition of one pro-inflammatory cytokine may lead to perturbed expression of another pro-inflammatory cytokine. Without understanding of the underlying mechanisms and defining the molecular predictors it may be difficult to control cytokine release that accompanies certain disease manifestations. Accumulating evidence suggests that in some cases a combined pharmacological inhibition of pathogenic cytokines, such as simultaneous blockade of IL-4 and IL-13 signaling, or blockade of upstream cytokines, such as TSLP, are more effective than single cytokine targeting. IL-6 and TNF are the important inflammatory mediators in the pathogenesis of asthma. Preliminary data suggests that combined pharmacological inhibition of TNF and IL-6 during asthma may be more efficient as compared to individual neutralization of these cytokines. Here we summarize recent findings in the field of anti-cytokine therapy of asthma and discuss immunological mechanisms by which simultaneous targeting of multiple cytokines as opposed to targeting of a single cytokine may improve disease outcomes.

## Introduction

Asthma is a heterogeneous disease characterized by chronic airway inflammation affecting almost 20% of population in some countries (1). Asthma is now considered as an umbrella term for diagnosis of distinct mechanistic pathways and clinical manifestations (2). Typically, patients with asthma develop various respiratory symptoms: shortness of breath, cough and chest tightness, but underlying molecular events driving the pathogenesis, as well as disease severity, may vary significantly.

**Figure 1:**
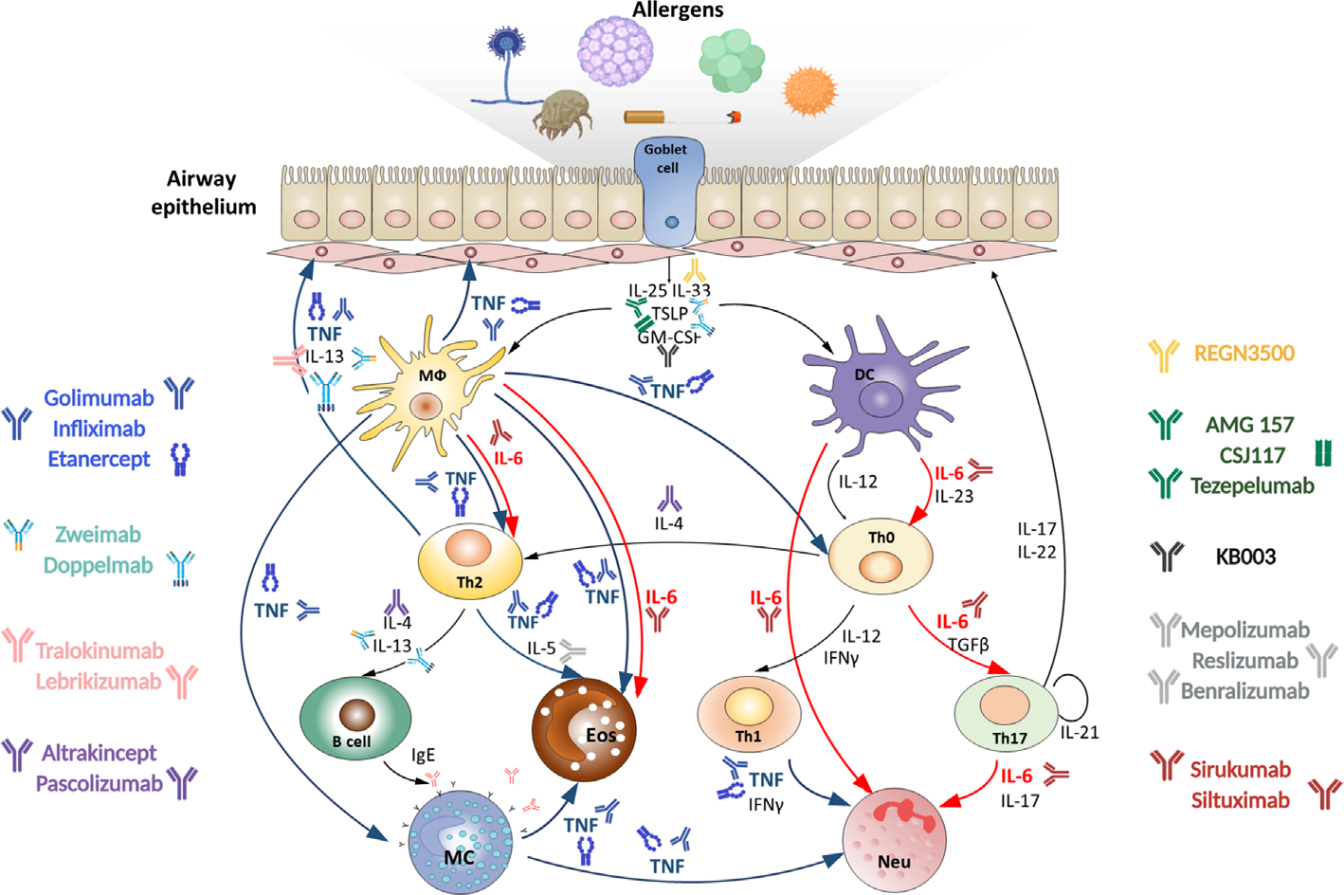
Targeting TNF, IL-6 and other cytokines in acute severe asthma. Severe asthma pathogenesis: epithelial cells exposed to pro‐inflammatory and allergic stimuli release mediators such as TSLP, IL-33 and IL-25, which activate dendritic cells. Within the airway lumen allergens can be captured by dendritic cells, which process antigenic molecules and present them to naïve (Th0) T helper cells. The consequent activation of allergen-specific Th2 cells is responsible for the production of IL-4 and IL-13 that promote B-cell operated synthesis of IgE antibodies and IL-5, which induces eosinophil maturation and survival. Eosinophils activated by IL-5 contribute to oxidative stress. On the other hand, in the presence of IL-23 and IL-6 upon dendritic cell activation Th0 cells differentiate into Th17 cells or into Th1 cells in the presence of IL-12. Th1 and Th17 cells stimulate and induce neutrophilic inflammation, characteristic of severe asthma. Combined pharmacological inhibition of TNF and IL-6 in acute allergic asthma may more effectively reduce the severity of inflammatory response in the lungs as compared to inhibition of these cytokines individually, since it blocks multiple pathogenic mechanisms. MФ, macrophage; DC, dendritic cell; Th, T helper; MC, mast cell; Eos, eosinophil; Neu, neutrophil.

Severe asthma is defined as a condition when adequate control of exacerbations cannot be achieved by treatment with high-dose corticosteroids and/or by standard therapy or if symptoms flare up when the treatment is reduced (3, 4). At the cellular level the thickening of basal membrane of respiratory epithelium and high granulocyte counts in sputum represent hallmarks of severe asthma (5). The disease progression is triggered by the activation of mucosal innate immune system. Resident antigen-presenting cells become activated in response to allergens and epithelial alarmins that are released due to the epithelial barrier disruption (6). The downstream mechanisms include activation of Th2/mast cell/eosinophil-mediated pathology, Th1/Th17/neutrophil-mediated pathology, chronic innate immune responses and irreversible airway obstruction. Studies in mice revealed that cytokines play an exceptional role in orchestrating each stage of severe asthma progression (**Figure 1**) (7). Th17- and Th2-mediated inflammatory pathways are regulated reciprocally during asthma (8), thus, inhibition of type 2 cytokines, such as IL-4 or IL-5, or administration of corticosteroids leads to reorganization of immune response from Th2-mediated eosinophilic to Th17-mediated neutrophilic disease (9, 10). The earliest signature cytokines of the epithelial damage, IL-33, TSLP (thymic stromal lymphopoietin) and IL-25, promote activation and migration of APCs (antigen-presenting cells), which, in turn, produce pro-inflammatory mediators with a broad spectrum of functions (ex. IL-6 and TNF). During the maturation of adaptive immunity, IL-4, IL-5, IL-13 and eotaxin are indispensable for the type 2 immune responses and are associated with differentiation of Th2 and ILC2 (type 2 innate lymphoid cell) subsets as well as with eosinophil migration (11). IL-17 and IL-6 are known regulators of Th17 immune responses and neutrophilia (12). Macrophage-expressed CXCL8 (chemokine ligand 8) fuels neutrophil accumulation and oxidative damage mediated by these cells (9). TGFβ (transforming growth factor beta), IL-22, IL-6 and TNF also contribute to tissue remodeling and fibrosis that are associated with the late stages of asthma progression (13–16). Repeated cycles of epithelial injury and inadequate immune activation lead to chronic inflammation in the lungs.

Irreversible bronchoalveolar tissue remodeling in severe asthma patients causes a decrease in respiratory functions and may lead to live-threatening consequences. Сorticosteroids are ineffective in severe asthma treatment, furthermore, they can prevent apoptosis of neutrophils and promote extracellular matrix production, thereby, exacerbating airway remodeling (17). Accumulating evidence suggests that pro-inflammatory cytokines and their receptors may represent promising targets for monoclonal antibody-based therapy. However, pathological mechanisms typically involve multiple cytokines with partly overlapping functions. The idea of combined anti-cytokine inhibition in severe asthma is currently drawing considerable attention. In the next section, we review the most promising clinical studies in this area and provide some perspective for this therapeutic approach.

## Alarmins as Targets for Asthma Therapy

Airway epithelial cells represent the first line of defense on the host’s mucosal surfaces. The interaction between airway epithelium and the environment is crucial for asthma development. Allergen-induced epithelial damage causes the release of epithelial alarmins - endogenous, constitutively expressed, chemotactic and immune activating proteins (18–20). TSLP, IL-33, GM-CSF (granulocyte-macrophage colony-stimulating factor) and IL-25 are the key airway epithelium-derived cytokines, acting like alarmins, which are released in response to the loss of epithelial cell integrity induced by allergens, proteases or viruses.

Release of TSLP, IL-33, IL-25 and GM-CSF is obviously not restricted to allergy; however, these cytokines mostly contribute to type 2 immune response through specific activation of dendritic cells. In particular, GM-CSF (21) and TSLP (22) overexpression in the lungs may induce spontaneous Th2 response to inhaled ovalbumin. IL-33 was shown to drives IL-1β-dependent Th2 inflammation in mice sensitized and challenged intranasally with ovalbumin and chitin (23), while IL-25 enhances eosinophil recruitment to the airways as well as goblet cell hyperplasia (24). Since epithelial alarmins constitute the first wave of signaling molecules released in response to allergen exposure, the idea of using them as potential therapeutic targets appears attractive.

The efficacy of anti-TSLP, anti-IL-33 and anti-IL-25 as monotherapies for treatment of allergic diseases was addressed in a number of studies. For example, in a mouse model of ovalbumin-induced asthma administration of anti-IL-33 antibodies was shown to decrease eosinophil infiltration, IgE production and Th2 cytokine release (25), as well as airway hyperreactivity (AHR) (26). Specific targeting ST2 (IL1RL1, Interleukin 1 receptor-like 1), an IL-33 receptor, demonstrated similar effects in ovalbumin-challenged mice (27). In a mouse model of persistent house dust mite (HDM)-induced asthma characterized by mixed granulocytic influx in the lungs the anti-IL-33 treatment was shown to prevent airway remodeling (28). Importantly, phase 2a randomized placebo-controlled study of anti-IL-33 in peanut-induced allergy suggests that a single dose of Etokimab, IL-33 blocker, could reduce atopy-related adverse effecs. Therefore, IL-33 targeting is a promising therapeutic strategy for allergic asthma (29) (**Table 1**).

**Table 1:** Ongoing clinical trials and clinical research of anti-cytokine therapeutic inhibitors.

Treatment/drug	Inhibitor type	Target	Phase	Asthma endotype	Status	Outcome	ID
***Ongoing trials***							
CSJ117	Inhaled FAB-fragment of human monoclonal antibody	TSLP	I	Mild atopic asthma	Completed	Reduction of allergen-induced bronchoconstriction	NCT03138811
Tezepelumab	Human monoclonal antibody	TSLP	II	Mast сell phenotype asthma and AHR	Recruiting		NCT02698501
			II	Severe asthma	Completed	FEV1 levels improve by 0.11–0.15 L more in Tezepelumab group than in placebo group. Blood eosinophil counts, FeNO, and total serum IgE levels decrease in Tezepelumab group	NCT02054130
			III	Severe asthma	Active, not recruiting		NCT03347279
			III	Severe asthma	Active, not recruiting		NCT03406078
REGN3500	Human monoclonal antibody	IL-33	I	Allergic asthma	Completed	Asthma control improvement. Blood eosinophil counts decrease	NCT03112577
Combination of REGN3500 and Dulimumab	Human monoclonal antibody	IL-33, IL-4Rα			Completed	Asthma control improvement. Blood eosinophil counts decrease. Combination of the two therapies failed to perform better than Dulimumab alone	
KB003	Human monoclonal antibody	GM-CSF	II	Moderate-to-severe asthma	Completed	FEV1 levels improve by 0.06–0.25 L more in KB003 group than in placebo group. No effects on asthma control or exacerbation rates	NCT01603277
Benralizumab	Humanized monoclonal antibody	IL-5	III	Severe eosinophilic asthma	Completed	Improved asthma control	NCT01928771
					Completed		NCT01914757
Golimumab	Human monoclonal antibody	TNF	II	Severe asthma	Completed	Provoking of serious adverse infections and malignancies	NCT00207740
Etanercept	Human dimeric p75 receptors fused with human Fc		II	Moderate to severe asthma	Completed	Improvement in lung function and quality of life	NCT00141791
			Not applicable	Refractory asthma	Completed	No positive effect	NCT00276029
Sirukumab	Human monoclonal antibody	IL-6	IIa	Severe uncontrolled asthma	Withdrawn	Withdrawn before participants were enrolled	NCT02794519
***Clinical research***							
Infliximab	Chimeric monoclonal antibody	TNF		Severe steroid-dependent asthma		Reduced frequency of asthma exacerbations	(30)
Tocilizumab	Humanized monoclonal antibody	IL-6R		Severe persistent asthma		Clinical and immunological responses to therapy. Suppression of IL-4^+^Foxp3^+^ (Th2-like) and IL-17^+^Foxp3^+^ (Th17-like) Treg cells, Th2- and Th17-cells	(31)

TSLP is a master-regulator of type 2 response at mucosal surfaces. In a mouse model of chronic HDM-induced allergic asthma (32) and in an ovalbumin-induced asthma both in rats (33) and in mice (34) neutralization of TSLP with monoclonal antibodies decreased Th2 cytokine levels and prevented structural alterations in the airways. Importantly, the efficacy of fully human anti-TSLP monoclonal antibody, AMG 157, was assessed in a small group of patients with mild atopic asthma (35). This double-blind placebo-controlled study revealed that three monthly doses of AMG 157 could reduce both allergen-induced bronchoconstriction and indexes of airway inflammation. Moreover, in a phase 2 trial addition of Tezepelumab, a TSLP blocking agent, resulted in attenuated asthma exacerbation rates in severely affected patients with uncontrolled asthma (36). Currently, two phase 3 trials for Tezepelumab are underway: the NCT03347279 trial will evaluate Tazepelumab in patients with uncontrolled asthma and the NCT03406078 trial will examine whether Tezepelumab treatment can reduce the required daily doses of oral corticosteroids in patients with severe asthma (**Table 1**). Finally, the development of inhalable high-affinity version of anti-TSLP blocking antibody (37) and the evaluation of its effectiveness (38) is under investigation.

IL-25, or IL-17E, is a member of the IL-17 family of cytokines, regulating type 2 immunity (39, 40). Administration of anti-IL-25 antibodies in mice with ovalbumin- (41) or HDM- and adenoviral smad2–overexpression-induced asthma (42) significantly decreased the Th2 immune responses and attenuated AHR and airway tissue remodeling. Clinical trials of anti-IL-25 efficacy for asthma treatment are yet to be conducted.

Targeting of alarmins proved beneficial as an add-on therapy or in combination with other cytokine inhibitors. Blockade of TSLP together with CRTH2, a chemoattractant receptor homologue expressed on Th2 memory cells, attenuated eosinophilic inflammation and AHR in ovalbumin-challenged mice (43). In line with this, bispecific anti-TSLP, anti-IL-13 reagent was recently developed in a form of monovalent antibody called Zweimab, and bivalent bispecific antibody scaffold – Doppelmab (44). However, further *in vivo* studies are required to evaluate their efficacy in asthma models.

Despite the substantial role of IL-33 in driving Th2-mediated responses, ST2 deficient mice are not resistant to allergic asthma (45), suggesting that inhibition of IL-33 alone may not be sufficient for preventing asthma development. The detailed analysis of allergic responses in ST2 knockout mice revealed the compensatory increase of TSLP production in response to allergen challenge (46). To test whether anti-IL-33 or anti-TSLP antibodies may attenuate inflammation, RAG1-/- (recombination activating gene 1 knockout) mice were intravenously sensitized by adoptively transferred ILC2 cells from immunocompetent mice and then intranasally challenged with eosinophil extracellular traps. It was found that anti-IL-33, but not anti-TSLP, reduced IL-5 and IL-13 production, while AHR was decreased only in anti-TSLP treated mice (47). The effect of broad neutralization of alarmin-mediated signaling in asthma was further evaluated in ST2-/- mice. Administration of anti-IL-25 and anti-TSLP antibodies during ovalbumin-induced asthma could reduce the infiltration of inflammatory cells into airways, as well as local expression of IL-4, IL-5 and IL-13. Moreover, attenuated airway tissue remodeling and histopathological features related to inflammation were observed under these experimental conditions (48). Interestingly, in a mouse model of influenza-induced exacerbation of allergic asthma only IL-33-specific neutralization resulted in improved AHR, prevented body weight loss and accumulation of inflammatory cells in the lungs, whereas combined administration of anti-TSLP and anti-IL-33 did not provide any additional benefits as compared to anti-IL-33 monotherapy (49). Nevertheless, taking into account the impressive results with anti-TSLP therapy in severe asthma, one may expect that in such case combined anti-IL-33 and anti-TSLP therapy could be even more beneficial. Taken together, these findings suggest that simultaneous targeting of multiple alarmins may provide synergistic therapeutic effects in asthma treatment.

## Targeting Th2 Allergic Responses With Multiple Agents

After the initial steps in allergic response and alarmin release, the effector cytokines typically come into play. IL-4, IL-5 and IL-13 are the central Th2 cytokines responsible for eosinophilic inflammation in asthma. IL-5 is required for the maturation and release of eosinophils from the bone marrow and for their tissue accumulation and activation, and, therefore, represents a relevant target for treatment of eosinophilic inflammation. Up to date, two anti-IL-5 antibodies Mepolizumab (50) and Reslizumab (51) are approved for severe eosinophilic asthma, and the third antibody, Benralizumab, has passed phase 3 trials. Post hoc analysis of pooled phase III SIROCCO (NCT01928771) and CALIMA (NCT01914757) data for patients with severe eosinophilic asthma confirmed that Benralizumab improved asthma control (52). Taking together, anti-IL-5 therapy is a valuable treatment option for patients with severe eosinophilic asthma.

Both IL-4 and IL-13 are Th2 cytokines, the aberrant production of which has long been associated with allergic disorders. IL-4 and IL-13 are encoded by adjacent genes (located on chr 5q in humans and chr11 in mice) that share a common regulatory element (GATA-3) and can transmit signals through a shared functional receptor complex IL-4Rα/IL-13Rα1, thus, not surprisingly, these two cytokines have many common functions (53). However, some of their activities appear to be non-redundant. For example, IL-4 predominantly contributes to eosinophilia and Th2 activation (53) but has no impact on airway remodeling and AHR, while IL-13 upregulation is sufficient to induce mucus production, bronchoconstriction and AHR (54). Neutralizing these two cytokines by a monoclonal antibody targeting their common receptor, represents the most impressive example of a multiple cytokine inhibition in asthma. While both anti-IL-13 antibodies, Tralokinumab (55, 56) and Lebrikizumab (57) and both anti-IL-4 antibodies, Altrakincept (58) and Pascolizumab (59), failed to provide beneficial effects for patients with asthma, blocking signal transduction through IL-4- and IL-13-shared receptor complex with Dupilumab, a fully human monoclonal antibody against IL-4Rα, is efficient and is now approved for treatment of severe asthma patients (60–62).

## TNF and IL-6 Neutralization in Asthma

Serum TNF represents an important biomarker for severe asthma (63). TNF-TNFR1 signaling in airway smooth muscle cells causes AHR (64, 65), supports chronic inflammation, as well as lymphocyte and granulocyte infiltration in the lungs (66, 67). Proliferation and transdifferentiation of fibroblasts are also dependent on TNF-induced expression of TGFβ (68). Therefore, blocking this cytokine in severe asthma initially appeared promising, and administration of anti-TNF reagents demonstrated encouraging results in animal asthma models. For example, in acute model of asthma, administration of monoclonal anti-TNF antibodies was associated with reduced inflammatory cell infiltration, airway goblet cell metaplasia and AHR (69, 70).

Anti-TNF biologics as therapeutics for severe asthma were tested in a number of clinical studies. However, Golimumab, a human monoclonal antibody, binding both soluble and transmembrane forms of TNF failed to demonstrate therapeutic effects in severe asthma; furthermore, patients experienced serious side effects (71). Systemic anti-TNF therapy is known to be associated with increased risk of infections and neoplasms (72, 73), and may affect the integrity of granulomas, which control *Mycobacterium tuberculosis* (74). Moreover, TNF blockade may lead to accumulation of Th17 cells and high levels of IL-17A production (75). On the other hand, Etanercept, a recombinant fusion protein based on TNF receptor 2, significantly improved lung function in patients with severe asthma and high TNF levels in the sputum (76, 77). In line with this, Infliximab, a chimeric monoclonal antibody, significantly decreased asthma exacerbations rate in patients with moderate and corticosteroid-resistant refractory asthma (30, 76, 78). Taken together, these findings suggest that TNF inhibitors remain promising therapeutic reagents for severe asthma, but inhibition of TNF alone may not be sufficient to fully control the disease pathology and also may lead to serious side effects, which are mediated by overexpression of other proinflammatory cytokines.

IL-6 is a pleiotropic cytokine involved both in regulation and induction of chronic inflammation (79, 80) with a wide range of cellular sources and complex receptor system (80–83). IL-6 determines the direction of CD4^+^ T cell differentiation (84, 85), suppresses the Treg cells, attracts myeloid cells to the site of inflammation and promotes fibrosis (86) in the absence of IL-13.

Increased levels of IL-6 were detected in serum, sputum, and bronchoalveolar lavage fluid (BALF) of asthmatic patients and correlated with disease severity (87, 88). The role of IL-6 from different cellular sources in the respiratory allergic pathology is being actively investigated. It was shown that IL-6 may shift differentiation of macrophages towards alternatively activated macrophages (M2), which play a key role in eosinophilic inflammation in HDM-induced asthma (89). Moreover, mice with IL-6 deficiency in macrophages showed significantly decreased eosinophilic inflammation in the lungs and reduced production of Th2-associated cytokines and IgE (89, 90), while IL-6 deficiency in dendritic cells resulted in decreased Th17-, but not Th2-inflammatory response (90). Thus, IL-6 produced by dendritic cells may contribute to the development of severe neutrophilic asthma. Taken together, IL-6 is implicated in progression, severity and duration of asthma and should be considered as a target for anti-cytokine asthma therapy.

Selective blockade of distinct modes of IL-6 signaling has different outcomes (91, 92). The IL-6 receptor complex includes IL-6R and gp130. The classical pathway is initiated by the interaction of IL-6 to membrane-bound IL-6R, then gp130, localized on the surface of the same cell, joins the complex. The trans-signaling pathway is mediated by a soluble IL-6R (sIL-6R), which may form as the result of alternative splicing or proteolytic shedding. Membrane bound IL-6R is present on the surface of a limited number of cell types, including leukocytes, whereas expression of gp130 is almost ubiquitous providing sensitivity to IL-6 to a broad range of cells upon IL-6/sIL6-R interaction (91). This type of signaling has pathogenic effects in several severe asthma endotypes (93). Both anti-IL-6 antibodies (Sirukumab, Siltuximab) and anti–IL-6R antibodies (Tocilizumab, Sarilumab) are able to block classical and trans-signaling pathways. The newly described third mode of IL-6 signaling – so called trans-presentation - which is involved in formation of pathogenic Th17 cells in EAE (experimental autoimmune encephalomyelitis) mouse model (94) can be inhibited by IL-6R blockers only (91), although the significance of that type of signal transduction in asthma pathogenesis is not yet established.

A clinical trial of Sirukumab, a human monoclonal antibody against IL-6, in severe asthma was withdrawn after FDA had disapproved Sirukumab (NCT02794519) for treatment of rheumatoid arthritis, due to increased number of deaths and malignancies among patients (95). On the contrary, Tocilizumab, a humanized anti-IL-6R monoclonal antibody, is being successfully used for treatment of chronic inflammatory diseases such as rheumatoid arthritis, systemic juvenile idiopathic arthritis and Castleman’s disease (96, 97). Recent case report study on severe pediatric persistent asthma showed clinical and immunological responses to Tocilizumab as an additional therapy without any adverse effects (31). Within 10 months on anti-IL-6R therapy patients showed a decrease in Th2 and Th17 cellular responses, however, peripheral eosinophilia in these patients was not impacted (31). Another clinical study in mild allergic asthma found no evidence that Tocilizumab is able to prevent allergen‐induced bronchoconstriction (98).

Thus, TNF neutralization may have a marked potential to improve lung functions, but demonstrates serious side effects, while blocking of IL-6 does not provide significant therapeutic effect in severe asthma. Interestingly, the simultaneous administration of both inhibitors prevented the increase of eosinophilic and neutrophilic infiltrate in BAL fluid in mouse model of acute HDM-induced asthma (unpublished data). Thus, our preliminary results indicate that combined pharmacological inhibition of TNF and IL-6 in acute allergic asthma may be more effective in reducing the severity of the inflammatory response in the lungs as compared to inhibition of these cytokines individually. More studies are needed to define minimally required doses of the two biologics and to address the issue of possible adverse effects under these conditions.

## Concluding Remarks

Effective and safe therapy of severe asthma remains an unresolved problem for modern medicine. Severe eosinophilic asthma is manageable due to FDA approved IL-4Rα blocker – Dupilumab, which affects the two main cytokines of Th2 immune response (IL-4 and IL-13) simultaneously (99). In addition, recent studies in mice revealed therapeutic potential of combined blockade of early markers of epithelial barrier damage – IL-33 and TSLP (44). The strategy for combined anti-cytokine therapy is aimed to overcome functional redundancy of pro-inflammatory cytokines. In this review we discussed the perspective of using combination of blockers to reduce both redundancy and the side effects of a monotherapy. IL-6 and TNF are key pro-inflammatory cytokines implicated in neutrophilia, extracellular matrix production and chronic inflammation during severe asthma. In spite of possible side effects, TNF neutralization is a golden standard in treating rheumatoid arthritis, psoriasis and inflammatory bowel disease therapy (100). Anti-IL-6 drugs are also approved in RA (101). In mice HDM-induced severe asthma a combination of TNF and IL-6 inhibitors prevented IL-6-dependent eosinophilia as IL-6 neutralization helped to suppress the pathological side effects associated with systemic TNF neutralization. Multiple cytokine targeting has demonstrated its efficacy in severe eosinophilic asthma. We propose that similar approach may help to find long-awaited treatment for severe neutrophilic asthma.

## Data Availability Statement

The raw data supporting the conclusions of this article will be made available by the authors, without undue reservation.

## Author Contributions

EGu, ON, EGo, AM, MD, and SN discussed the concept and wrote the manuscript. All authors contributed to the article and approved the submitted version.

## Funding

This work was supported by RSF grant №19-75-30032.

## Conflict of Interest

The authors declare that the research was conducted in the absence of any commercial or financial relationships that could be construed as a potential conflict of interest.
